# Novel Polymer-Free Everolimus-Eluting Stent Fabricated using Femtosecond Laser Improves Re-endothelialization and Anti-inflammation

**DOI:** 10.1038/s41598-018-25629-9

**Published:** 2018-05-09

**Authors:** In-Ho Bae, Myung Ho Jeong, Kyung Seob Lim, Dae Sung Park, Jae Won Shim, Jun-Kyu Park, Kwang Hwan Oh, Mi Rim Jin, Doo Sun Sim

**Affiliations:** 1 Cardiovascular Convergence Research Center of Chonnam National University Hospital Designated by Korea Ministry of Health and Welfare, Gwangju, 61469 Republic of Korea; 2 Korea Cardiovascular Stent Research Institute, Jangsung, 57248 Republic of Korea; 3 Department of Cardiology, Chonnam National University Hospital, Gwangju, 61469 Republic of Korea; 4 Research Institute of Medical Sciences, Chonnam National University, Gwangju, 61186 Republic of Korea; 5 Laser Advanced System Industrialization Center, Jeonnam Technopark, Stiftung, Jangsung 57248 Republic of Korea

## Abstract

The aim of this study was to fabricate a novel polymer-free everolimus-eluting stent with nanostructure using a femtosecond laser (FSL). The stent were coated with everolimus (EVL) using FSL and electrospinning processes. The surface was rendered hydrophobic, which negatively affected both platelet adhesion (82.1%) and smooth muscle cell response. Animal study was performed using a porcine coronary restenosis model. The study groups were divided into 1) bare metal stent (BMS), 2) poly(L-lactide) (PLA)-based EVL drug eluting stent (DES), 3) commercial EVL-eluting DES, and 4) FSL-EVL-DES. After four weeks of stent implantation, various analyses were performed. Quantitative analysis showed that the amount of in-stent restenosis was higher in the BMS group (BMS; 27.8 ± 2.68%, PLA-based DES; 12.2 ± 0.57%, commercial DES; 9.8 ± 0.28%, and FSL-DES; 9.3 ± 0.25%, *n* = 10, *p* < 0.05). Specifically, the inflammation score was reduced in the FSL-DES group (1.9 ± 0.39, *n* = 10, *p* < 0.05). The increment in re-endothelialization in the FSL-DES group was confirmed by immunofluorescence analysis. Taken together, the novel polymer-free EVL-eluting stent fabricated using FSL can be an innovative DES with reduced risk of ISR, thrombosis, and inflammation.

## Introduction

Atherosclerosis is the primary cause of coronary artery disease, which contributes to high mortality rates in modern society. A drug-eluting stent (DES) is a useful tool that remarkably reduces stenosis rate and has been widely used as an intervention in cardiology in the past decade^1,2^. However, certain limitations associated with DES are yet to be overcome. Especially, the use of polymers for coating drugs onto bare metal stent (BMS) surfaces is related to severe adverse effects such as inflammation and thrombosis^3,4^. Moreover, polymer degradation may form fragments, which can cause embolism^5,6^. In addition, several problems should be considered prior to using polymers, such as the generation of surface non-uniformity by cracking or peeling, mechanical change by strut bridging, and occlusion in peripheral vessel by bulk erosion^7–9^. To overcome these limitations, the innovation of a special surface morphology for anchoring drugs to the stent-surface of polymer-free DES is critical. Post drug elution, a DES is exposed to the blood and the environment. Thus, biophysical surface modification and optimization have become one of the most attractive research areas in biomaterial science^10,11^. The application of femtosecond laser (FSL) pulses in medicine is a growing field of interest; for example, FSL is currently used in refractive surgery for vision correction^12^, neurosurgery^13^ or nanosurgery in single cells^14^. The main advantage of using FSL pulses is the high cutting precision in the micrometer range accompanied by minimal collateral damage. Moreover, reports show that cell migration and organization can be directed via laser patterning^15^. Despite the advantages of FSL, such as the ability to regulate cellular processes and accumulation of data regarding the utility of FSL-based medical devices, data on surface modification of stents is scarce. Previously, we have reported that a protein can be incorporated inside the nanoscaled pores on the surfaces of medical devices by a simple loading process^16^. Everolimus (EVL), a derivative of sirolimus that acts as an inhibitor of the mammalian target of rapamycin (mTOR), is commonly coated onto BMS. Owing to its ability to reduce restenosis, it has been extensively studied and widely used in patients with coronary artery diseases^17–19^. The aim of this study was to fabricate a novel polymer-free DES with nanopatterns and nanopores using FSL. The cellular response regulatory ability of the nanopatterns and drug storage capacity of the nanopores were evaluated. Furthermore, the feasibility of FSL was verified by investigating the alteration of mechanical properties post-FSL and in a preclinical animal study.

## Results

### Morphological analysis of femtosecond laser-treated stent

**Figure 1:**
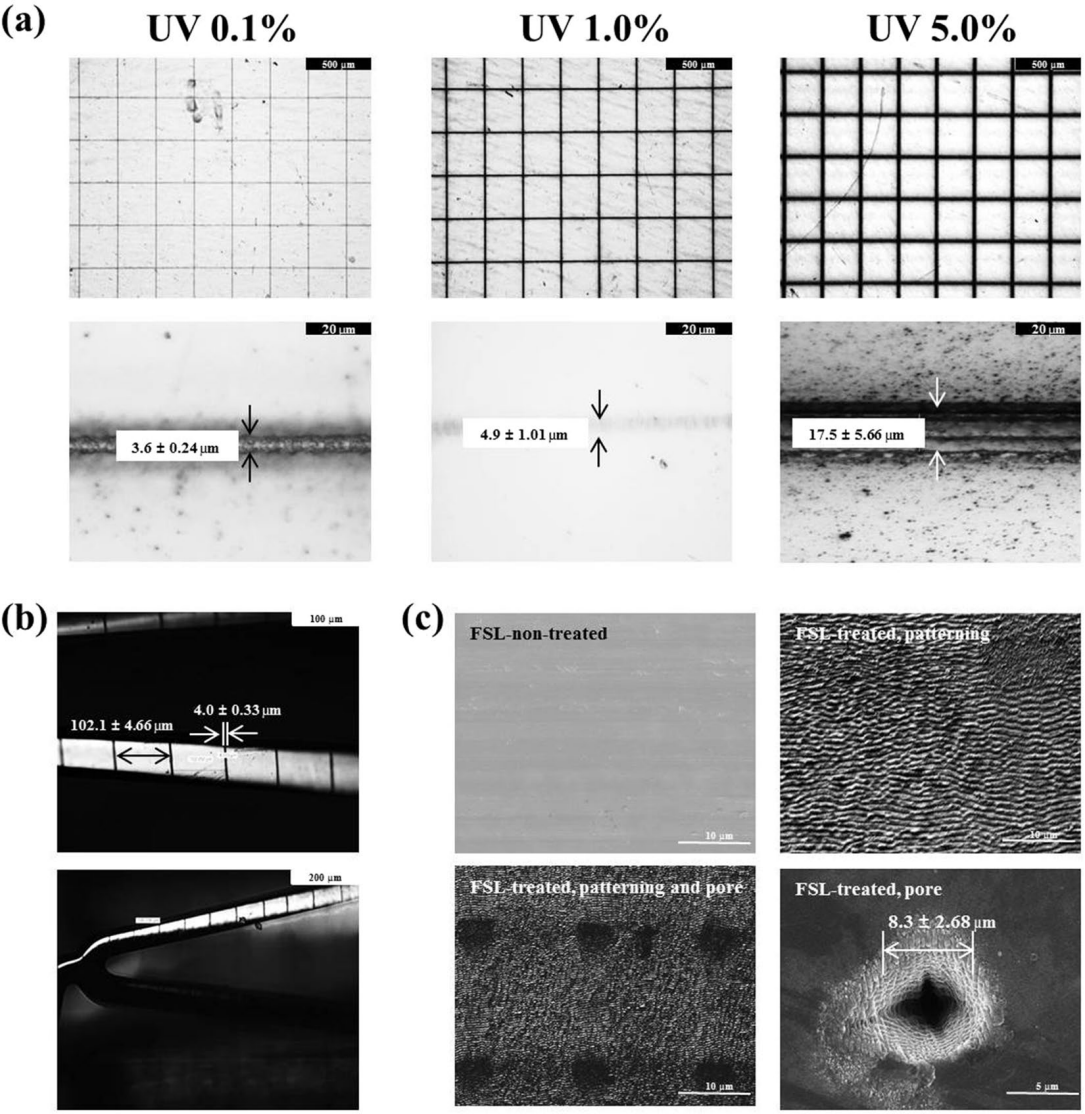
Morphological analysis after femtosecond laser irradiation. Representative SEM images of surfaces 2-D plates (**a**) and 3-D tubular stents (**b**) under various irradiation conditions. Representative images of patterning and pores on stent surfaces after femtosecond laser irradiation (**c**).

The surface morphologies of the stents were investigated using OM and SEM. The thickness of the patterned line that underwent FSL irradiation was regulated by controlling the UV intensity (Fig. 1a). The round shape of the plates (15 × 15 mm) was utilized for this study. The thickness gradually increased at higher UV intensity (3.6 ± 0.24 μm in 0.1% UV group, 4.9 ± 1.01 μm in 1.0% UV group, and 17.5 ± 5.66 μm in 5.0% UV group, respectively, n = 10). We selected 1.0% UV for further study since it showed the high thickness without any FSL-induced metal damage. As shown in Fig. S1, we designed the rotating system so as to enable FSL irradiation of a 3-dimensional (3-D) tubular stent. The 3-D stent was irradiated with FSL using conditions identical to those of the plate study. Although the thickness of the patterned line was less in the 3-D stent study (4.0 ± 0.33 μm, n = 10) than in the plate study, the difference was not significant. The intervals between lines were approximately 102.1 ± 4.66 μm (Fig. 1b). After FSL treatment, the surface morphology was rough and wavy. Pores were created using FSL irradiation by focusing the laser, and the diameter was approximately 8.3 ± 2.68 μm (Fig. 1c). Unfortunately, it was hard to obtain appropriate images of the pores in the 3-D tubular study (data not shown).

### Mechanical performance study of femtosecond laser irradiation

**Figure 2:**
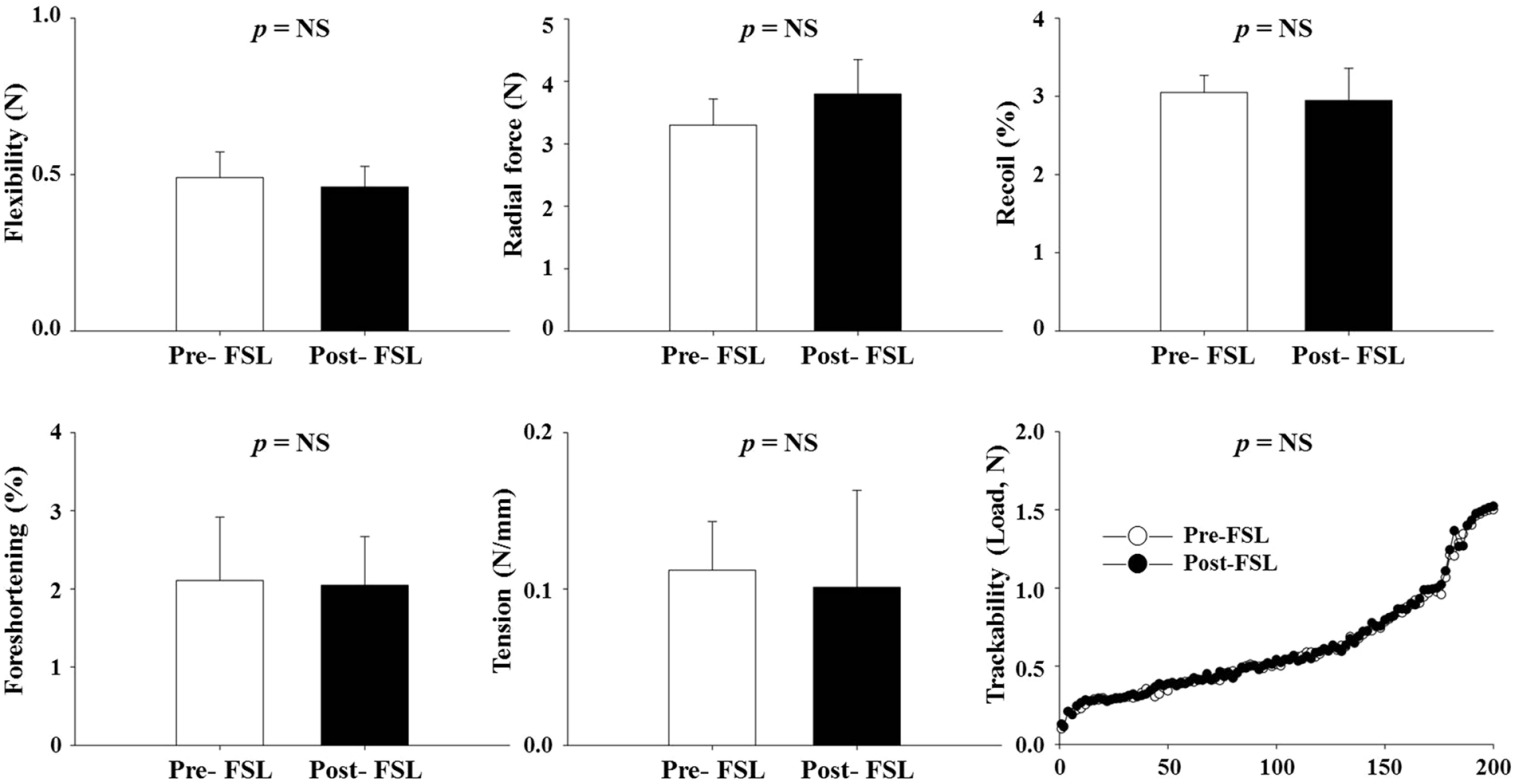
Mechanical properties of stent pre- and post-femtosecond laser irradiation. The indicated values are expressed as mean ± SD (n = 10). NS, not statistically significant.

The finite element method (Fig. S2) and various mechanical tests were performed under the KTL guideline to investigate the variations in the mechanical properties of the stent after FSL irradiation. Results show that the mechanical performance did not change after FSL irradiation (pre-; 0.49 ± 0.082 N vs. post-FSL; 0.46 ± 0.066 N in flexibility, pre-; 3.3 ± 0.42 N vs. post-FSL; 3.8 ± 0.55 N in radial force, pre-; 3.05 ± 0.220% vs. post-FSL; 2.95 ± 0.415% in recoil, pre-; 2.11 ± 0.810% vs. post-FSL; 2.05 ± 0.620% in foreshortening, and pre-; 0.12 ± 0.031 N/mm vs. post-FSL; 0.10 ± 0.062 N/mm in tension, respectively, n = 10, *p* = ns). Trackability analysis was performed to investigate the accessibility of the stent to lesions. Results show that FSL did not change the accessibility (Fig. 2).

### Contact angle measurement

**Figure 3:**
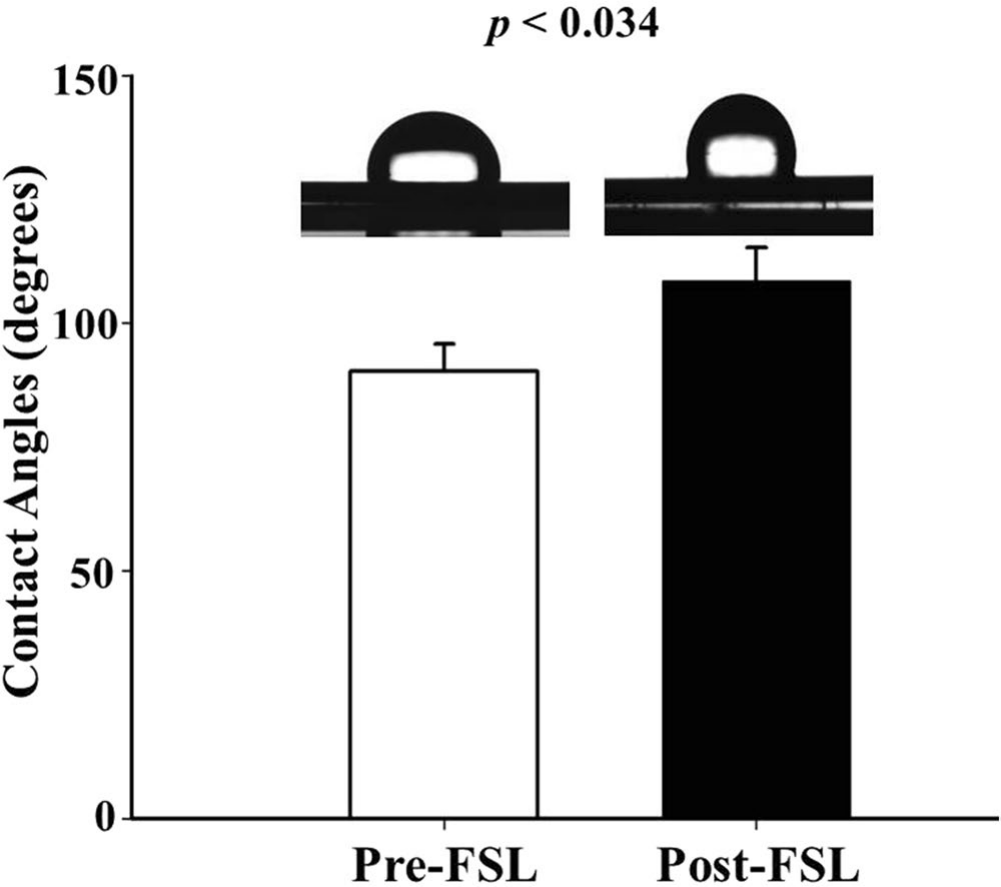
Measurements of surface wettability. Images of surface static contact angles of the stents were represented and analyzed. The indicated values are expressed as the mean ± SD (n = 10).

The surface property of FSL was investigated by contact angle measurement. The contact angles measured at the left and the right edges of the water drop were almost symmetrical. The contact angle was more significantly increased in the post-FSL group (108.5 ± 6.82°, 20.2%) than in the pre-FSL group (90.3 ± 5.50°, n = 10, *p* = 0.034), indicating that the FSL process improved hydrophobicity (Figs 3 and S3).

### Cellular response by femtosecond laser irradiation

**Figure 4:**
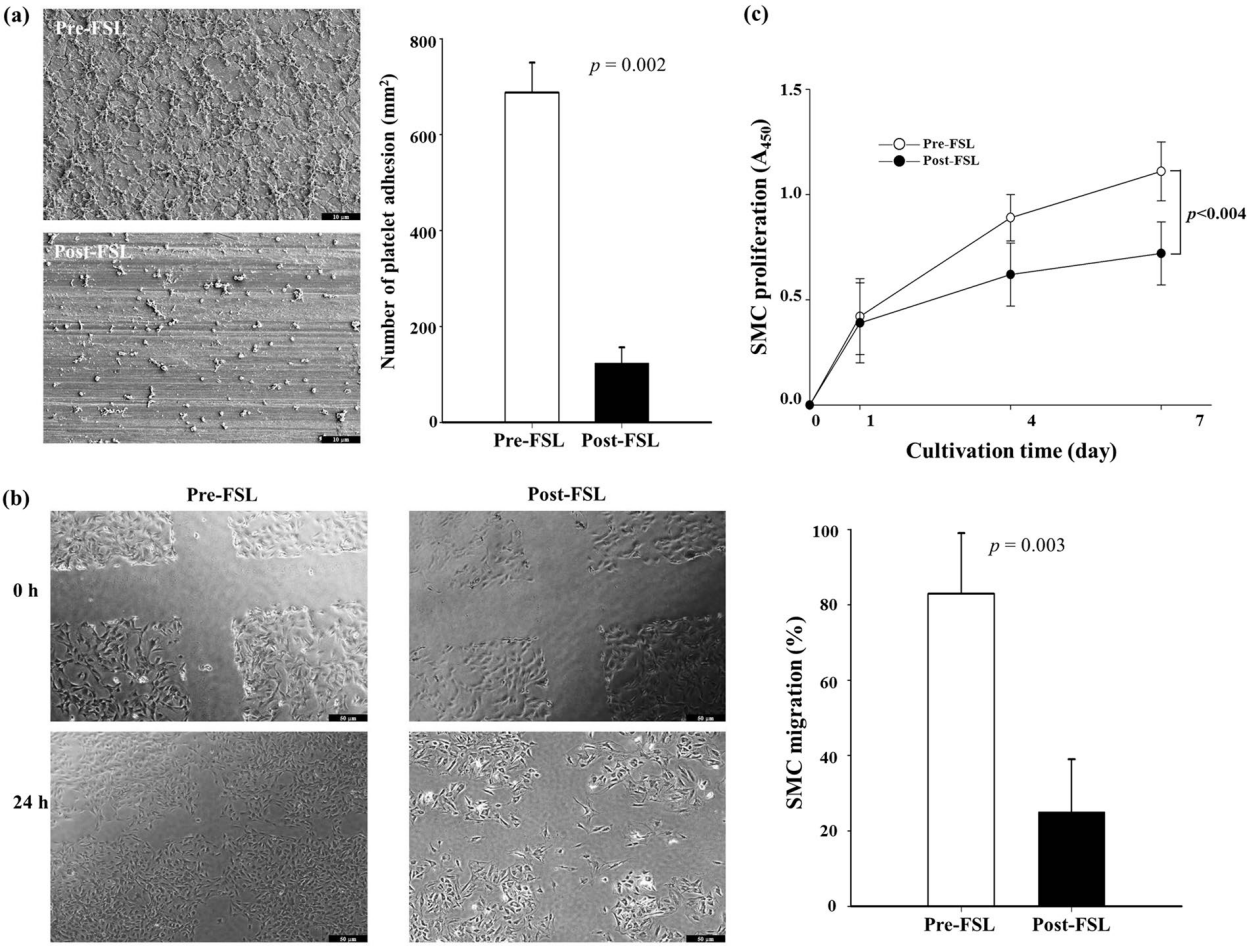
Platelet adhesion and smooth muscle cell response after femtosecond laser irradiation. Representative images and image analysis of platelet adhesion (**a**) and SMC migration (**b**) XTT analysis for SMC proliferation. (**c**) The indicated values are expressed as the mean ± SD (n = 10).

We assessed the adhesion and distribution of platelets on the FSL-treated surfaces by calculating the number of blood platelets. The number of blood platelets on the post-FSL surface (123 ± 33/mm^2^, 82.1%) was clearly lesser than that on the pre-FSL surface (688 ± 62/mm^2^, n = 10, *p* = 0.002) under the same conditions (Fig. 4a). The extent of SMC migration was investigated to determine cellular response after FSL. SMCs migrated to the scratched area (12.0 ± 1.05 mm^2^) after 24 h of post-scratched cultivation time in the FSL non-treated group (10.8 ± 4.21 mm^2^, 90.1 ± 8.70%). In contrast, the SMC migration rate was reduced in the post-FSL group (9.0 ± 3.88 mm^2^, 23.0 ± 7.45%; Fig. 4b, n = 10, *p* = 0.003). Further, the XTT assay was performed to investigate the inhibitory effect of FSL. SMC proliferation was inhibited more in the post-FSL group (0.71 ± 0.073 at 7 days, 33.0 ± 4.13%) than in the control group (1.06 ± 0.062; Fig. 4c, n = 10, *p* < 0.004).

### Measurement of coating amount and release velocity of everolimus

**Figure 5:**
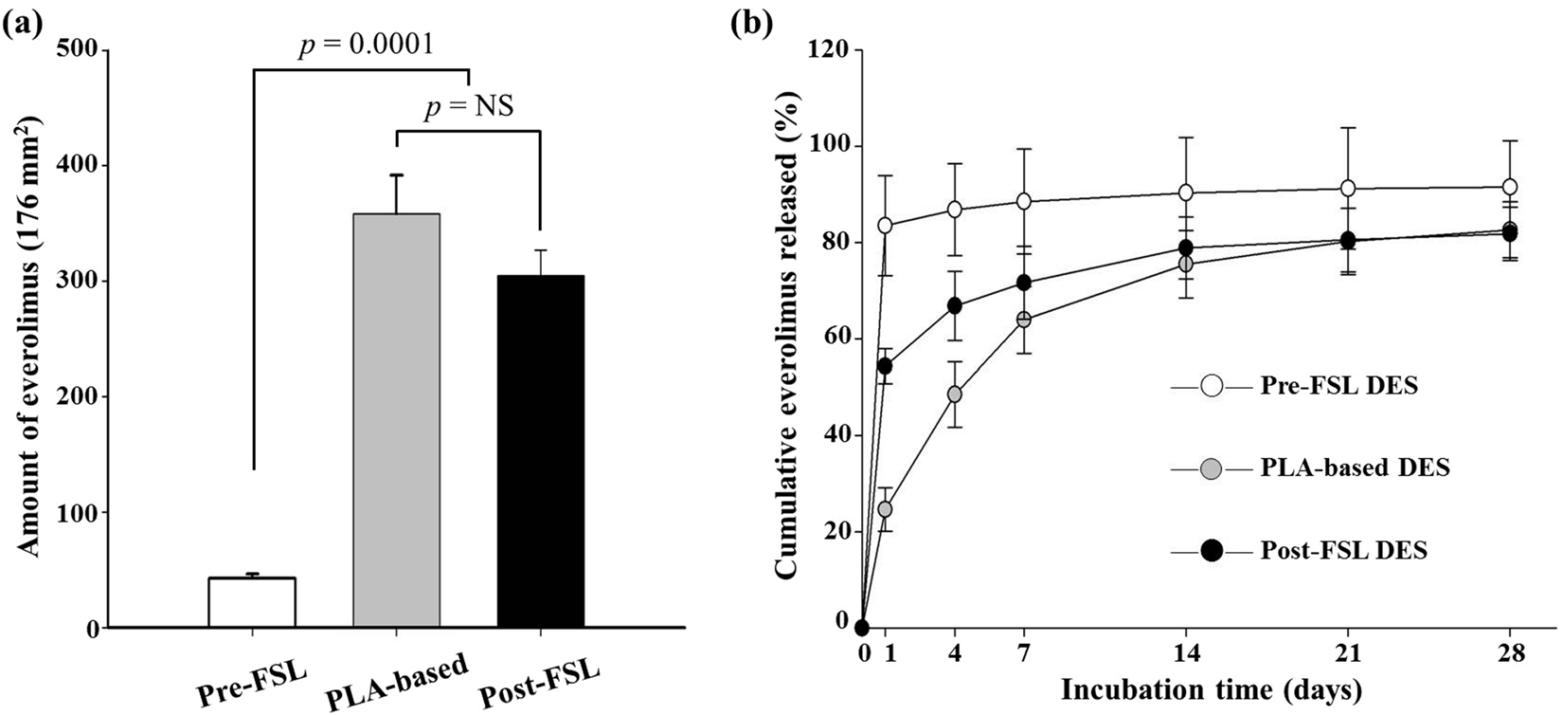
Amount of everolimus on surfaces and *in vitro* release velocities. The amount of EVL on the stent surfaces after washing with PBS. (**a**) *In vitro* cumulative EVL released from surfaces. (**b**) The amount of everolimus was measured using UV-visible spectrophotometer at designated time points. The indicated values are expressed as the mean ± SD (n = 10). NS, not statistically significant.

EVL was coated on the tubular stent and plates using an electrospinning machine (Fig. S4). For comparison, bare and PLA-based plates were subjected to electrospinning under same conditions as described above. The amount of EVL on the plate surfaces were expressed as cumulative EVL concentrations. Results show that the amount of EVL on the pre-FSL plate surface was negligible (42.5 ± 3.85 µg/176 mm^2^). In contrast, the amount of EVL on the post-FSL plate (304.5 ± 22.18 µg/176 mm^2^), n = 10, *p* = 0.0001) was considerably higher than that in pre-FSL plate. The amount of EVL on the post-FSL plate was slightly less than that on the PLA-based EVL coated group (358.4 ± 33.28 µg/176 mm^2^, n = 10, *p* = 0.048) (Fig. 5a). The amount of EVL released from plate surfaces was measured as mentioned above. Results showed a burst-out pattern in the pre-FSL DES group (83.5 ± 10.45% in 1 day). On the contrary, the EVL release velocity was significantly slower in the post-FSL group, which enabled interpretation of a non-burst out pattern (54.3 ± 3.62% in 1 day and 66.8 ± 7.23% in 4 day). Although slightly faster than the release velocity observed with the PLA-based DES group, the amount of EVL released from stents were similar in both groups at 7 days of incubation (63.9 ± 6.92% in PLA-based DES and 71.6 ± 7.64% in post-FSL DES, respectively) (Fig. 5b).

### Analysis of animal study – nondestructive analysis

**Figure 6:**
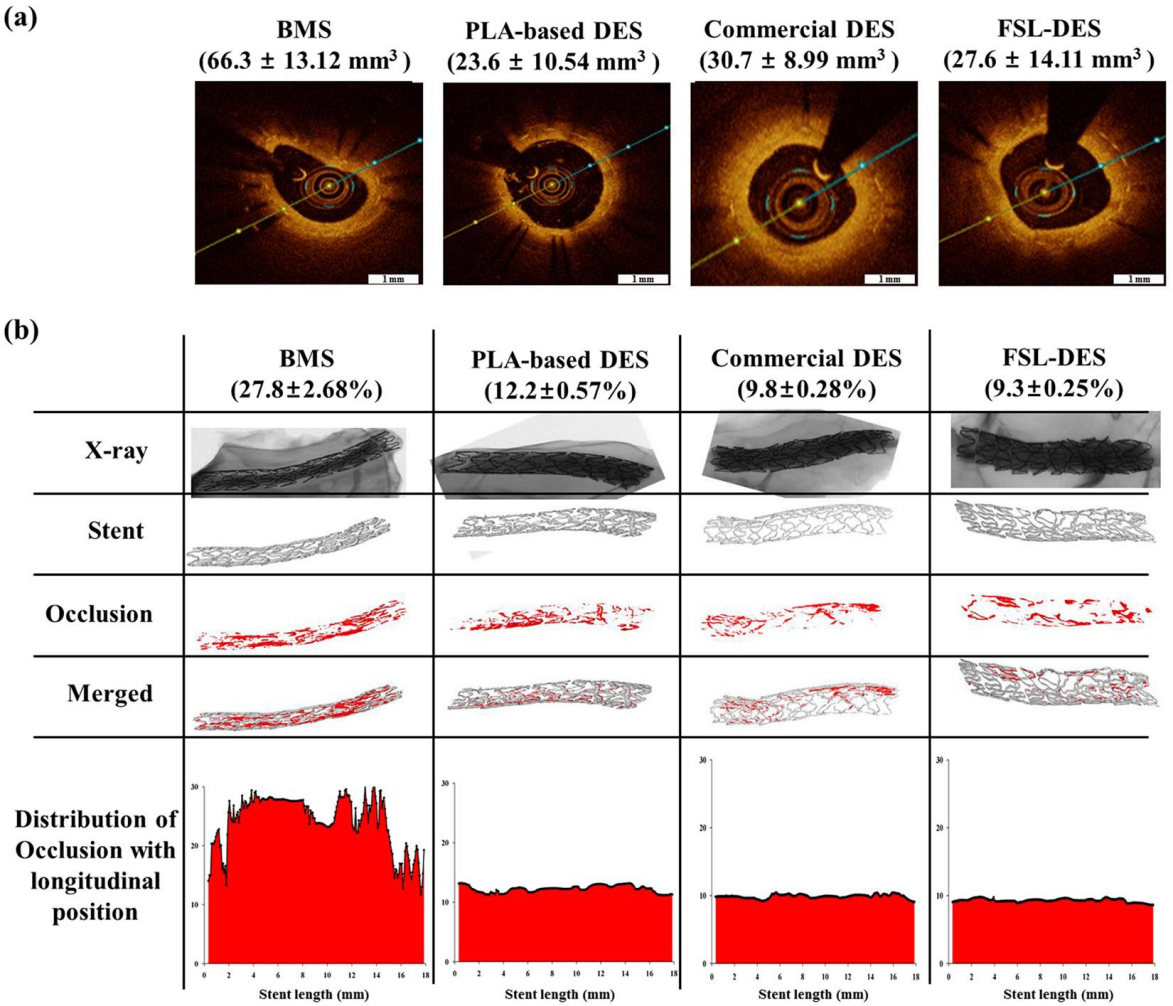
Quantitative analysis of animal study. After 4 weeks of implantation, the vessels surrounding stents were isolated and subjected to OCT (**a**) and microCT (**b**) analysis. Representative images of each was shown (n  = 10).

To verify the results of the *in vitro* experiments, pre-clinical animal study was performed using a porcine coronary restenosis model. BMS, PLA-based DES, commercial EVL-eluting DES, and FSL-EVL-DES were randomly implanted in the left anterior descending coronary artery and left circumflex artery with stent:artery ratio of 1.3. Four weeks after stent implantation, vessels surrounding the stents were isolated and fixed in 10% neutral-buffered formalin. Prior to histopathological analysis, nondestructive analysis such as OCT and microCT analysis was performed. OCT results show that the NIH area was reduced in the EVL-containing group (PLA-based DES; 23.6 ± 10.54 mm^3^, commercial DES; 30.7 ± 8.99 mm^3^, and FSL-DES; 27.6 ± 14.11 mm^3^, respectively. n = 10) compared to that in the BMS group (66.3 ± 13.12 mm^3^) (Fig. 6a). These tendencies were corroborated by the microCT results (BMS; 27.8 ± 2.68%, PLA-based DES; 12.2 ± 0.57%, commercial DES; 9.8 ± 0.28%, and FSL-DES; 9.3 ± 0.25%, respectively, n = 10). The distribution of occlusion with longitudinal position of microCT indicated mild type-2 ISR (Fig. 6b).

### Analysis of animal study – histological analysis

**Figure 7:**
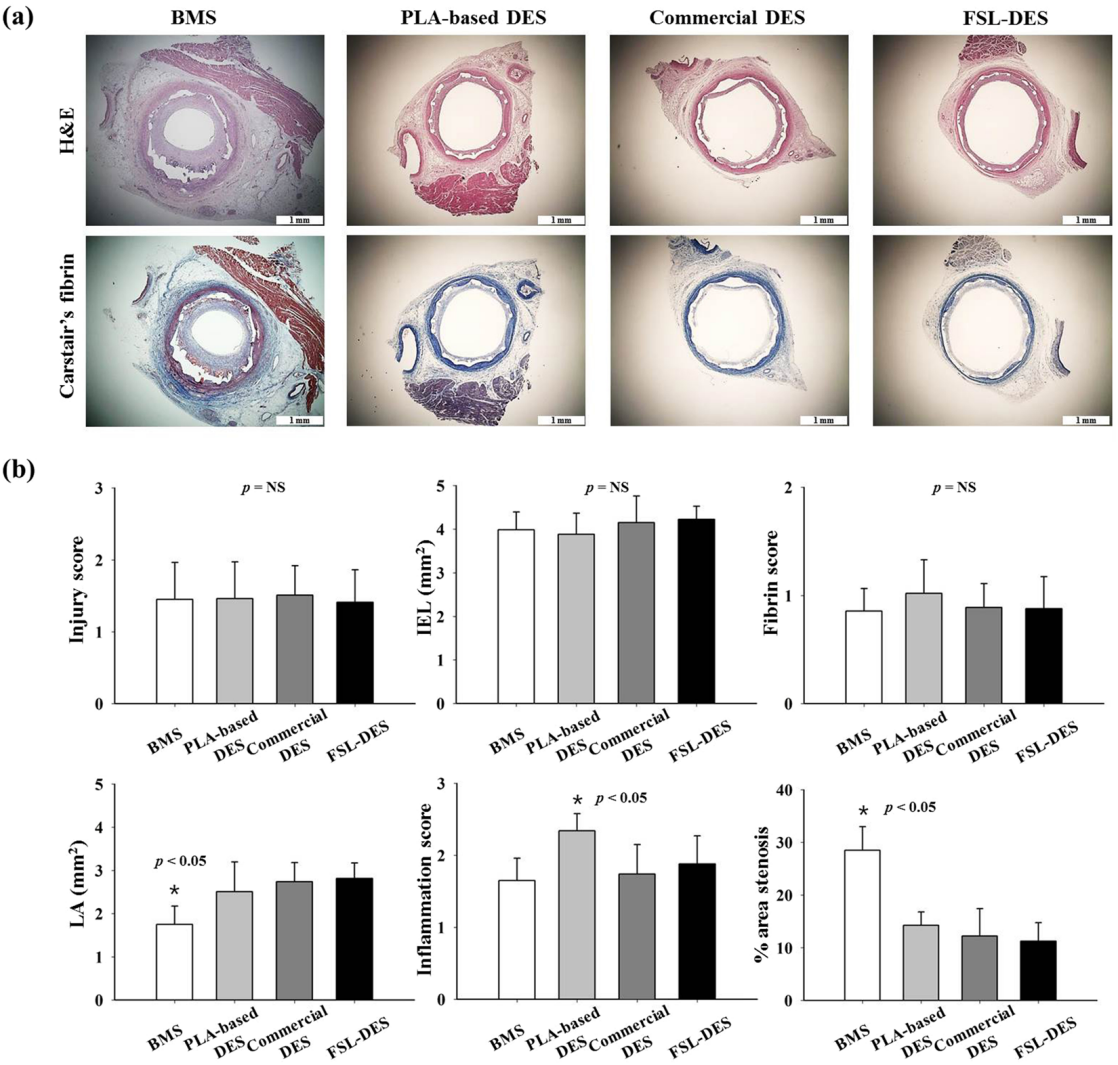
Histological analysis of the porcine coronary restenosis model. After 4 weeks of implantation, the vessels surrounding stents were isolated and subjected to H&E and Carstair’s fibrin staining. Representative images of sections (**a**) and histomorphometric analyses (**b**) are shown. Magnifications of the cross-sectional slices were 25×. (n = 10), NS, not statistically significant.

After OCT and microCT analysis, the same samples were subjected to histological analysis. There were no significant differences in the injury score (BMS; 1.5 ± 0.51, PLA-based DES; 1.5 ± 0.51, commercial DES; 1.5 ± 0.41, and FSL-DES; 1.4 ± 0.45, respectively, n = 10, *p* = NS), IEL (BMS; 4.0 ± 0.41, PLA-based DES; 3.9 ± 0.49, commercial DES; 4.2 ± 0.61, and FSL-DES; 4.2 ± 0.30, respectively, n = 10, *p* = NS), and fibrin score (BMS; 0.86 ± 0.21, PLA-based DES; 1.02 ± 0.31, commercial DES; 0.89 ± 0.22, and FSL-DES; 0.88 ± 0.29, respectively, n  = 10, *p* = NS). However, the LA was significantly lower in the BMS group (1.75 ± 0.42) compared to that of other groups (PLA-based DES; 2.51 ± 0.68, commercial DES; 2.74 ± 0.44, and FSL-DES; 2.81 ± 0.36, respectively, *n* = 10, *p* < 0.05). Considering the lower value of LA in the BMS group, the percentage area of stenosis was higher (28.5 ± 4.5%) than in the other groups (PLA-based DES; 14.3 ± 2.55, commercial DES; 12.2 ± 5.2, and FSL-DES; 11.3 ± 3.50, respectively, n = 10, *p* < 0.05). Especially, the inflammation score was significantly higher in the PLA-based DES group (2.3 ± 0.24) compared to other groups (BMS; 1.6 ± 0.31, commercial DES; 1.7 ± 0.41, and FSL-DES; 1.9 ± 0.39, respectively, n = 10, *p* < 0.05) (Fig. 7a,b).

## Discussion

In past decades, synthetic polymer-based everolimus-eluting DES have been widely used for effective treatment of obstructive coronary artery disease and prevention of ISR that is mainly caused by hyperproliferation of smooth muscle cells^20^. However, DES has been replaced with BMS to eliminate drug release after full polymer degradation from the stent surface. Thereby, the BMS is exposed to a physiological environment as a foreign material^21^. Especially, synthetic polymer-related inflammation and thrombosis remain major limitations, and surface properties have to be modified to overcome these challenges^22,23^. Especially, it is well-known that the acidic products generated during polymer degradation (*i.e*., lactic acid and glycolic acid) may elicit inflammatory responses in the vessel wall^24^. Several histological studies show that inflammatory reactions play a pivotal role in the proliferation of neointimal hyperplasia and restenosis after stent implantation, which support the results of this report^25,26^. Therefore, in this study, we developed novel polymer-free everolimus-eluting stent using the FSL irradiation technique. Previously, we have reported that nanopores on metal surfaces are able to store and deliver drugs to the targeted tissue^16^. EVL anchoring by physical methods such as electrospinning, negative pressure, and lyophilization enable the exclusion of polymer during DES fabrication. First, we prepared equipment suitable for FSL irradiation of 3-D tubular shaped stents (Fig. S1). In such an equipment system, by adjusting the condition of the jig that is being held, the interval of the desired patterning can be realized. This equipment enabled regulation of pattern thickness and creation of micropores (Fig. 1), indicating the feasibility of directing cell migration^15^ and EVL storage^16^ without any critical variation in the mechanical properties of the CNUH stent (Figs 2 and S2). Clinical studies have also suggested that restenosis is correlated with the geometric properties of stents^27^. These geometric properties are important factors in biomechanical determinations and may affect stenting techniques^28^. Therefore, it is essential to investigate changes in the physical properties of the CNUH stent with optimal conditions established by the FSL process. Preferably, surface property was rendered hydrophobic after processing for FSL (Figs 3 and S3). The relationship between microstructure and hydrophobicity has been well documented^29,30^. As shown in Fig. 1c, the formation of microstructure by FSL in this study seems to be a factor that increases the hydropolicity of the surface. Coronary stent implantation, which is a foreign matter in the human body, may lead to in-stent thrombosis. Therefore, the first event in blood-biomaterial interaction is the adsorption of proteins onto the surface of the materials. Proteins such as fibrinogen, von Willebrand factor (vWF), albumin, and fibronectin rapidly adsorb onto the surface when a foreign material comes in contact with blood. The adsorbed protein layer determines all further events such as platelet adhesion, aggregation, and coagulation. It is well known that platelet adhesion is the primary cause of thrombosis. Therefore, platelet adhesion tendency was assessed to determine homocompatibility of the stent. The number of blood platelets on the post-FSL surfaces was lesser than that on pre-FSL BMS surface under the same conditions (Fig. 4). The anti-thrombogenic effect of FSL-irradiated surface results from its hydrophobicity, which may inhibit SMC migration and proliferation, the primary cause of ISR. The interaction of cells with biomaterials is of key importance for the successful long-term implantation of medical devices. Previously, it was reported that microstructured surface modification through femtosecond processing can regulate cell migration to specific regions^15^. Microstructure-related surface hydrophobicity affects the cell response^29^. In this study, cell proliferation and migration were also significantly suppressed on the microstructure surface by femtosecond processing. Electrospinning was used for EVL coating (Fig. S4). The smooth surface of BMS was disabled to anchor EVL (i.e. wash out by low outside force), and the anchoring, capturing, and delivering capacities of EVL-coated stents were greatly increased only with FSL process, negative pressure, and lyophilization (Fig. 5). The EVL release velocity was similar to that of sirolimus^31^. Pre-clinical animal study was performed in porcine coronary restenosis model to verify the *in vitro* results. There were four study groups. The BMS group was a negative control to compare the variation after FSL and drug-coating (i.e. DES). The PLA-based DES group was a control for the non-polymer stent to determine the effect of the synthetic polymer to compare the synthetic polymer-based DES to the non-polymeric FSL-based DES under our same processing conditions. The commercial DES group was a positive control to compare the performance of the FSL-DES with that of products widely used in the market. Finally, the FSL-DES group comprised the final product in this study. The stents were implanted in the porcine coronary arteries and the stent-surrounding vessels were isolated at 4 weeks post-implantation and subjected to histopathological analysis (using immunofluorescence) and quantitative analysis (using OCT and microCT). Angiographic images were obtained prior to sacrificing the animals. The images showed the positions of the properly-expanded implanted stents and that blood was passing freely through the lumens of the implanted stents (Fig. S5). OCT and microCT analysis showed that occlusion was lower in all EVL-containing groups, but not in BMS (Fig. 6). These values were consistent with LA and percentage area stenosis obtained from histological observation (Fig. 7). Previous reports show that acidic degradation induces inflammation^32^. As expected, the inflammation score was significantly higher in the PLA-based DES group than in other groups. The FSL-DES was fabricated only with patterning since the 3-D rotating system was disabled to create pores. However, the animal study suggested that EVL was delivered to vessels by the nanopatterned stent. Further studies are warranted for analyzing the effect of patterning on drug storage and delivery. In conclusion, the novel polymer-free EVL-eluting stent fabricated using FSL can be an innovative DES with reduced risk of ISR, thrombosis, and inflammation.

## Methods

### Preparation of the bare metal stent

Previously, we designed a coronary BMS called CNUH (Chonnam National University Hospital) stent^33^, which was approved by the Korean Food and Drug Administration. CNUH stent showed superior flexibility and biocompatibility, which are important features of a good coronary stent. The CNUH stent was fabricated under identical manufacturing conditions as reported previously^34,35^. Briefly, cobalt-chromium alloy (Co-Cr, L605, 3.0 × 18.0 mm with tubular shape and 15 × 15 mm with round shape plate) was used as a stent material because several studies have demonstrated the biocompatibility of Co-Cr^36^. A laser cutter (Rofin, Starcut, Hamburg, Germany) was used for the fabrication of the BMS with the Co-Cr alloy. The BMS was exposed to an acidic atmosphere (50% H_2_SO_4_) for 1 h to remove burrs, and heat treatment and polishing were performed to restore the mechanical properties. Thereafter, the CNUH was subjected to FSL irradiation.

### Femtosecond laser irradiation process

The specimens were subjected to FSL irradiation to create nanoscale topographical patterns and pores (Supplementary Fig. S1). An ultra-short FSL with a maximum power of 6 W and center wavelength of 1,030 nm was used as the light source to direct patterned microgroove arrays on the work piece. The 2 mm-diameter laser beam was expanded five times using a beam expander and then tightly focused on the surface of the work piece with an objective lens (5×, focal length = 40 mm, working distance = 37.5 mm, numerical aperture = 0.14, depth of focus = 14 µm) after passing a linear polarizer and a quarter wave plate for optical isolation and polarization control. The laser spot diameter at the focus is calculated using the Gaussian beam equation, *d* = 4*fλ*_0_*/nπD*, where *f* is the focal length of the objective lens, *λ*_0_ is the laser beam wavelength, and *n* is the refractive index of air. Using *n* = 1 (air) and *D*  = 11.3 mm, the laser spot diameter was estimated to be approximately 4.6 µm and this value is used as the representative spot diameter throughout this study. The beam was circularly polarized at the surface of work piece to avoid a possible polarization dependence of the patterning process^37^. The direct-writing of the pattern was performed by translating the work piece using a high resolution (0.5 µm/pulse) air-bearing X-Y stage and motorized Z stage with the resolution of 1 m/pulse. The work piece was placed on a vacuum chamber that was installed on the X-Y-θ stage. All patterning processes were monitored in real time with a charge-coupled-device camera. Machining results were examined using an optical microscope and 3-dimensional surface profiler after ultrasonically cleaning of the samples.

### Surface morphology and properties

Optical microscopy (OM) was used to study the sample surface morphologies and for visual identification of color and deformation. Detailed morphologies such as patterning and pores were examined by scanning electron microscopy (SEM, Hitachi, Tokyo, Japan) with an acceleration voltage of 5 kV. The samples were dried overnight and sputter-coated using gold prior to SEM observation. The hydrophilicity of the stent surface was determined from static contact angle measurements of deionized water droplets. The degree of dispersion of 5 µL deionized water drops on the surfaces was measured with a contact angle meter (SEO300A, SEO, Suwon, Korea). For contact angle measurement, a round-shaped Co-Cr plate was used. Each data point represents an average of at least 10 independent measurements.

### Study of mechanical performance

To investigate the variation in the mechanical properties of the stent after FSL, the flat plate compression and 3-point bending tests, which provide radial force and flexibility measurements, respectively, were performed. Furthermore, we conducted foreshortening and recoil tests using simple mathematical equations to obtain the length and radius values of the stent before and after expansion. All mechanical performance studies, including a trackability, which is accessibility of the stent to lesions, were performed at the Korea Testing Laboratory (KTL) as reported previously^33^. A large deformation analysis was performed using the ABAQUS commercial code (Hibbit Karlsson & Sorenses Inc., Pawtucket, RI, USA) based on the finite element method.

### *In vitro* cellular response

To verify the effect of FSL on smooth muscle cell (SMC) proliferation, which are involved in stent restenosis (ISR), XTT analysis was performed at 1, 4, and 7 days of cultivation. Briefly, a 40 µL EZ-Cytox reagent (Daeil Lab Service Co., Seoul, Korea) was added to a 24-well culture dish. XTT is metabolized by mitochondrial dehydrogenases to form a formazan dye that can be spectrophotometrically determined by measuring the absorbance at 450 nm with a spectrophotometric microplate reader (Bio-Tek Instruments, Winooski, VT, USA). The amount of formazan salt formed corresponds to the number of viable cells in each well. A scratch assay for cell migration was performed to verify the results of the XTT assay. As we reported previously^35^, SMC (1 × 10^4^ cells/cm^2^) were seeded in 12-well plates containing pre-FSL and post-FSL disk. After 24 h of incubation, a line, 50 µm in thickness, was created by scraping through the center of the cell monolayer with a sterile tip. After 24 h of additional incubation at 37 °C in a humidified 5% CO_2_ atmosphere, fields of the scratched areas were randomly selected for imaging^38^. Cell migration was calculated as 100 × [(initial scratched area - remaining scratched area at 24 h incubation)/initial scratched area]. To estimate the thrombogenicity of the metal surface after FSL, a platelet adhesion test, which assesses hemocompatibility, was performed as reported previously^39^. Briefly, platelet-rich plasma (PRP) was obtained from fresh porcine whole blood by centrifuging blood with a 5 mL 3.0 wt% solution of sodium citrate at 150 × *g* for 15 min and 500 × *g* for 20 min. The PRP was harvested carefully and diluted to 3.0 × 10^7^ platelets/mL. The PRP solution was loaded onto the metal surfaces and allowed to rest at room temperature for 180 min. Thereafter, the non-specifically adhered PRP was removed by gently rinsing the surface. The samples were dehydrated with a concentration gradient of ethanol. Thereafter, the plates were subjected to SEM observation.

### Everolimus coating

EVL (20 mg/mL) was dissolved in tetrahydrofuran (THF) and coated onto the BMS and the plate using an electrospinning machine (ESB200, NanoNC, Seoul, Korea) under optimized conditions (5 kV voltage, 10 kgf/cm^2^ air pressure, 15 cm distance from the nozzle to the stent, 50 rpm rotation speed of the stent, and 60 µL/min spray speed). Thereafter, it was rotated with a homogenizer to prevent run-down of the EVL solution in a 37 °C dry oven for 30 min. Then, the samples were lyophilized under negative pressure. For the comparison group, poly(L-lactide) (PLA) (*Mn*  =  50,000 g/mol, Sigma Aldrich, USA) (20 mg/mL) and EVL (20 mg/mL) were dissolved in tetrahydrofuran (THF, Duksan Chemical, Seoul, KOR) and coated on the BMS surface.

### Determination of loading efficiency and release velocity of everolimus

The total amount of EVL on the plate surface before and after FSL was estimated. One hundred microliters of EVL solution (10 mg/mL) was carefully loaded onto the plates and incubated for 24 h to instill EVL on the surfaces. After gently washing the surface with de-ionized water to remove simple laid EVL from the surface, the plates were lyophilized as reported previously^40,41^. To measure the amount of EVL on the plates, the plates were immersed in THF solution with gentle shaking. The THF solution was then analyzed using an ultraviolet (UV) spectrophotometer at 278 nm. This measurement was continued till UV value was zero. The UV absorbance values were used to plot the EVL standard curve and cumulated. To determine the release velocity of EVL from the plates, the lyophilized plates were placed in phosphate-buffered saline (PBS, pH 7.2) solution. The PBS solution was taken out at every designated day and the absorbance was measured using the UV-visible spectrophotometer. The concentration of drug released was calculated by comparing it to the drug standard curve, and it was expressed in a cumulative manner.

### Animal preparation and stent implantation

All animal experimental procedures were performed in accordance with the The Ethics Committee of the Chonnam National University Medical School guidelines and regulations and all protocols were approved by Chonnam National University Hospital. Animal studies were performed with castrated male pigs weighing 20–25 kg. Study groups were divided into four groups: BMS, PLA-based DES, commercial EVL-eluting DES, and FSL-EVL-DES. The stents (40 stents) were randomly implanted in the left anterior descending coronary artery and left circumflex artery with a stent:artery ratio of 1.3. Four weeks after stent implantation, the animals were sacrificed by injecting 20 mL potassium chloride through the left carotid artery. Vessels surrounding the stents were isolated and fixed in 10% neutral-buffered formalin.

### Optical coherence and micro computed tomography analysis

Optical coherence tomography (OCT) images were acquired using a non-occlusive technique, through a 2.7 Fr C7 Dragonfly Imaging Catheter (LightLab Imaging Inc., Westford, MA, USA) that was flushed with undiluted contrast dye. Post-calibration, the catheter was inserted distal to the lesion of interest. Mechanical pullback at a speed of 20 mm/s was started during continuous automatic flushing of iodixanol (Visipaque™ 320 mg I/mL, GE Healthcare, Amersham, UK) at the rate of 2–5 mL/s, using a Medrad injector (Medrad Inc., Warrendale, PA, USA) to ensure blood clearance from the coronary arteries. Quantitative measurements were obtained using the neointimal hyperplasia (NIH) area (stent area–lumen area) (automatically traced and manually adjusted when required) as reported previously^42^. Prior to histological analysis, the isolated samples were subjected to micro-computed tomography (microCT; SKYSCAN 1172, Kontich, Belgium) analysis to quantify and visualize the ISR as reported previously^35,43^. Prior to scanning, the contrast media was injected into the lumen of the stent to highlight differences in the CT values of the lumen, strut, and occlusion. The samples were placed on microCT specimen table and scanned at 50 kV and 200 µA, with an exposure time of 1.2 s. The images were obtained by CTAN software (SKYSCAN, Kontich, Belgium). The ISR rate was calculated by subtracting the lumen extent from the stent extent. The distributions of occlusions were represented as a histogram.

### Histopathological analysis

Histopathological evaluation of the arteries was performed by an experienced cardiovascular pathologist. The specimens were embedded and 50–100-µm-thick sections were obtained at approximately 1 mm distance and subjected to hematoxylin-eosin (H&E) and Carstairs’ fibrin staining for histological analysis. Histopathological sections were measured using a calibrated microscope, digital video imaging system, and microcomputer program (Visus 2000 Visual Image Analysis System, IMT Tech, San Diego, CA, USA). Borders were manually traced for the lumen area (LA), area circumscribed by the internal elastic lamina (IEL), and innermost border of the external elastic lamina. Morphometric analysis of the neointimal area for a given vessel was calculated as the measured IEL area minus the LA. Measurements were obtained from five cross-sections from the proximal and distal ends, and three midpoints of each stent. The histopathological restenosis area was calculated as 100 × (1 − (lesion LA/lesion IEL area)^44^.

### Immunostaining assay

Immunocytochemistry (ICC) and immunofluorescence (IF) were conducted as previously described^45^. All samples were sectioned at 4 µm intervals, analyzed, and quantified with integral calculus. Representative images were selected from the mid-region of the stents. Serial sections of paraffin-embedded tissue were rehydrated by serially immersing them in xylene, alcohol, and water, washing with PBS and 0.1% Triton X-100, and then microwaving for 20 min in citrate buffer at pH 6.0 (Abcam, Cambridge, UK) for antigen retrieval. Sections were then blocked with bovine serum albumin (BSA, Sigma Aldrich). Primary antibody (1) mouse anti-smooth muscle actin antibody (Dako, 1A4), (2) rabbit anti-CD31 polyclonal antibody (Bioss, MA, USA), (3) anti-CD68 antibody (ab125212, Abcam) were diluted 1:400 in 0.05% Triton X-100. The sections were incubated overnight at 4 °C. Afterward, the sections were incubated with the respective Alexa Fluor 488 goat anti-rabbit IgG (green color) or 568 (red color) diluted in PBS at 1:400 and then washed with PBS. Sudan-block B at 0.1% was applied for 30 minutes and washed with 0.02% Tween 20 in PBS. After washed with PBS, 4′,6-diamidino-2-phenylindole (DAPI) staining was performed for 4 h at 4 °C. Slides were imaged through bright-field microscopy with a Nikon Eclipse E600 and a SPOT RT digital camera with accompanying software (Diagnostic Instruments). Excised stents were stored in formaldehyde solution. A 1.5 mL eppendorf tube was filled with clay, and the clay was molded into a V shape to hold the stent during contrast agent staining. The formaldehyde-fixed stents were placed vertically in the V-shaped opening in the clay. Each stent was positioned in the clay so that there was no movement of the stent inside the eppendorf tube. One milliliter of contrast agent (Omnihexol) was injected through the opening at the center of the stent with a 5 mL syringe.

### Statistical analysis

Statistical analysis was performed using commercially available software (SPSS version 15, Chicago, IL, USA). The data are presented as the mean ± SD. Unpaired Student’s *t*-test was used to compare the stent groups.

## Electronic supplementary material

Fig S1

Fig S2

Fig S3

Fig S4

Fig S5

Fig S6
