# Supplementary figures and images for: Novel Polymer-Free Everolimus-Eluting Stent Fabricated using Femtosecond Laser Improves Re-endothelialization and Anti-inflammation
Source: Sci Rep. 2018 May 9;8:7383. doi: 10.1038/s41598-018-25629-9 (PMC5943357; doi:10.1038/s41598-018-25629-9)

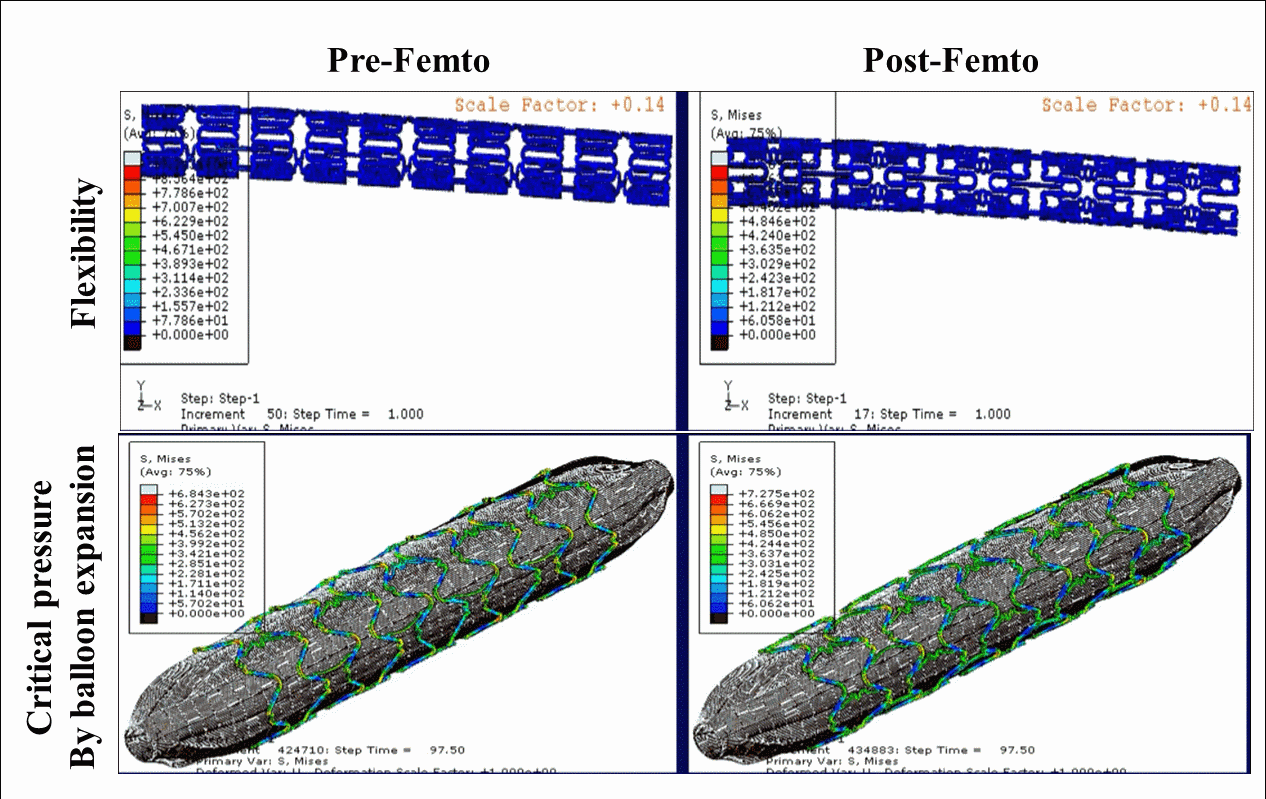

Supplement: Supplementary file 2 — Fig S2 [file 41598_2018_25629_MOESM2_ESM.gif]

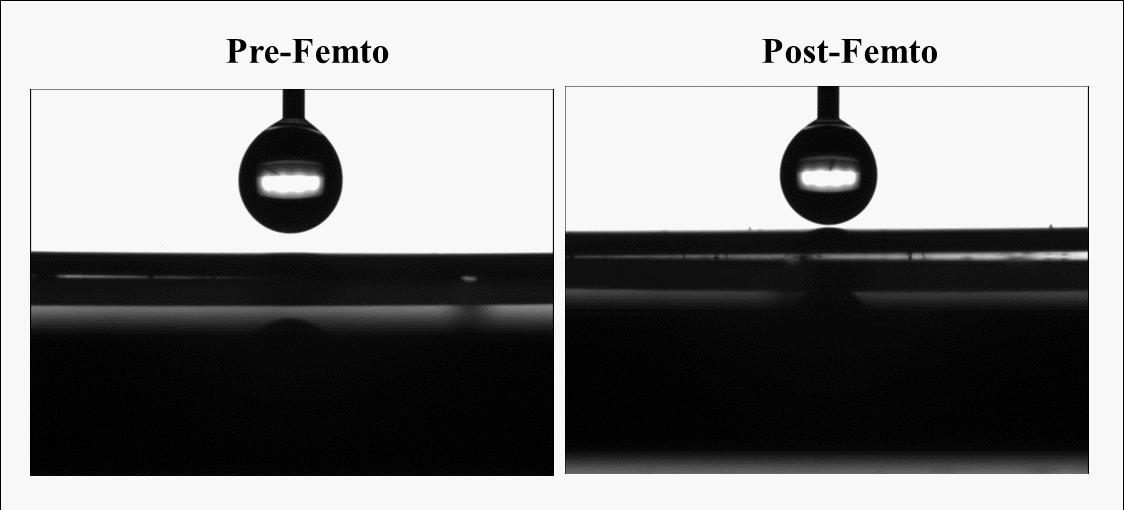

Supplement: Supplementary file 3 — Fig S3 [file 41598_2018_25629_MOESM3_ESM.gif]
